# DPMAS in the Management of Severe Acute Liver Injury

**DOI:** 10.1155/crhe/6456187

**Published:** 2026-01-13

**Authors:** Maiko Alejandro Tavera Díaz, Annia Aguilar Loayza, Carolina Beatriz Mejía Vargas, Juan Fernando Mamani Ochoa

**Affiliations:** ^1^ Department of Nephrology, Univalle Hospital, Cochabamba, Bolivia; ^2^ Department of Medicine, Univalle University, Cochabamba, Bolivia; ^3^ Department of Pathology, Elizabeth Seton Hospital, Cochabamba, Bolivia; ^4^ Department of Nursing, Elizabeth Seton Hospital, Cochabamba, Bolivia

## Abstract

**Background:**

Acute liver injury is a severe disease in which a hepatic and later systemic inflammatory response is triggered, generally induced by paracetamol intoxication, undetermined causes, drugs, and hepatotropic and nonhepatotropic viruses.

**Case Summary:**

A 67‐year‐old immunocompetent male with severe acute liver injury secondary to *Cytomegalovirus* (CMV) infection presented with a 2‐week history of anorexia, asthenia, adynamia, generalized weakness, myalgia, and jaundice. Laboratory tests revealed hyperbilirubinemia, hypertransaminasemia, coagulopathy, and acute kidney injury, and tests for hepatitis A, B, C, HIV, and autoimmune hepatitis were negative, while PCR was positive for CMV. Patient was treated with N‐acetylcysteine, albumin, valganciclovir, and liver support therapy using the dual plasma molecular adsorption system (DPMAS). Fundus examination showed CMV retinitis, and liver biopsy confirmed acute hepatitis with CMV cytopathic changes and areas of hepatocyte regeneration. After 5 sessions of DPMAS and 4 weeks of antiviral therapy, he showed clinical and biochemical improvement with native liver recovery and was discharged with outpatient follow‐up.

**Conclusion:**

This case highlights the successful early recognition and prompt initiation of appropriate treatment using antiviral therapy and liver support therapy in managing severe CMV‐induced acute liver injury in an immunocompetent patient, potentially averting the need for liver transplantation.

## 1. Introduction

Severe acute liver injury represents a phenotype of liver dysfunction characterized by an increase in transaminases up to 10 times their normal value, total bilirubin > 3 mg/dL, and coagulopathy (INR > 1.5), without developing encephalopathy. Non‐APAP etiology (induced by drugs other than paracetamol) has a worse prognosis, evolving to acute liver failure (ALF) with the probability of requiring liver transplantation and risk of death [1, 2]. Hepatitis and severe liver injury by nonhepatotropic virus due to *Cytomegalovirus* (CMV) in immunocompetent individuals is uncommon, whose clinical manifestations are malaise, fever, asthenia, adynamia, headache, myalgia, splenomegaly, hepatomegaly, adenomegaly, and pulmonary and/or digestive involvement [3–5]. Severe acute liver injury may progress to ALF and develop multiorgan failure [4, 6].

## 2. Case Presentation

**Figure 1:**
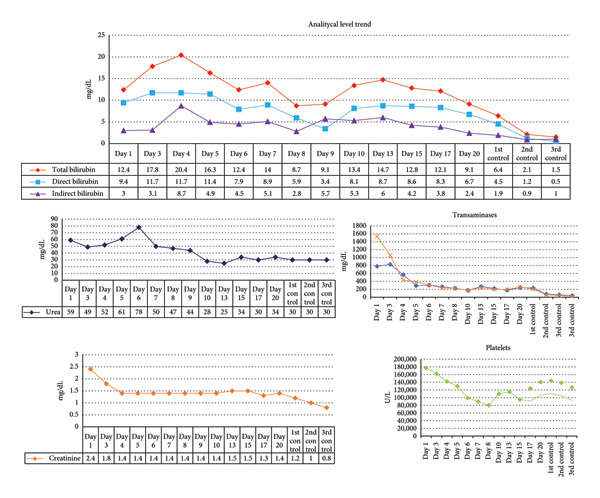
Temporal evolution of analytical tests during hospitalization and subsequent controls.

A 67‐year‐old male patient with a history of hypertension and dyslipidemia, with no previous liver involvement, attended the hospital emergency department with clinical symptoms of 2 weeks of evolution characterized by anorexia, asthenia, adynamia, generalized weakness, myalgia, and jaundice on skin and mucous membranes. On admission, the patient’s vital signs were stable. Physical examination revealed an alert patient who was globally oriented, without motor deficit or meningeal signs. Asterixis was negative, and reflexes were preserved. Jaundice was evident on skin and mucous membranes (+++); the rest of the examination was unremarkable.Laboratory tests showed hyperbilirubinemia, hypertransaminasemia, hyperazotemia (Figure 1), and coagulopathy (INR 2.0; PT 21.5 s). Monocyte count was 13.8%. Serological tests were negative for anti‐HAV IgM, anti‐HAV IgG, HBsAg, and anti‐HBc IgM. Anti‐HCV and ‐HIV were nonreactive. The autoimmune hepatitis panel (anti‐DNA, anti‐ANA, AMA, ASMA, and LKM1) was negative. PCR results for nonhepatotropic viruses (EBV, Parvovirus B19) were negative. CMV viremia was confirmed by quantitative PCR showing 12,000 copies/mL (4.08 log_10_ copies/mL). The quantitative CMV PCR had been requested on the seventh day of hospitalization, after ruling out other infections, autoimmune, and vascular causes; the result was obtained 2 days later, after liver support therapy sessions had already been initiated. No abnormality was found in the rest of the analyses. Based on the laboratory values (bilirubin, INR, and creatinine), the calculated Model for End Stage Liver Disease (MELD) score was 32 points. The patient evolved during hospitalization with deterioration of liver function, coagulopathy, and azotemia (Figure 1). He received treatment with N‐acetylcysteine as per protocol to limit oxidative stress and albumin at 1 mg/kg for management of acute kidney injury in the context of liver injury. Subsequently, the control laboratory showed a decrease in platelets and an increase in bilirubin values; this alteration is explained by platelet sequestration in hepatic sinusoids and a cause of perpetuation of liver injury and persistence of the inflammatory response. At 72 h after admission, given the ongoing deterioration of liver and kidney function, regardless of the cause and as a bridge therapy while awaiting etiologic confirmation and potential recovery, liver support therapy in the dual plasma molecular adsorption system (DPMAS) modality was performed to limit the inflammatory response, and 5 sessions were carried out with clinical and analytical improvement with a decrease of more than 50% in total bilirubin (Figure 1). Oral valganciclovir was started immediately after confirmation of CMV infection, with a planned total duration of 21 days (9 days completed during hospitalization and the remainder as an outpatient), given the unavailability of ganciclovir in our setting.

**Figure 2:**
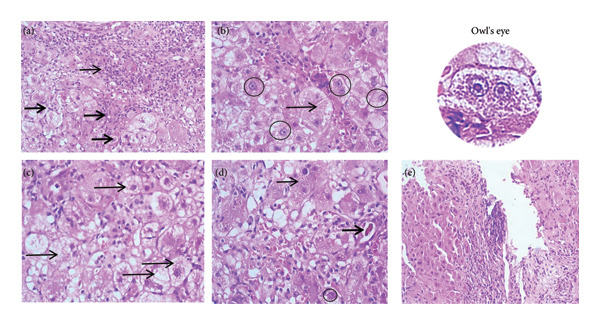
Histopathology of the liver. (a) Dense inflammatory infiltrate expanding the portal spaces and exceeding the limiting plate (thin arrow). The infiltrate, predominantly lymphocytic with neutrophils and eosinophils, is also present in sinusoidal spaces (thick arrows). Mild interstitial fibrosis is observed. H‐E, 20x. (b) Hepatocytes showing cytomegalovirus cytopathic effect with characteristic nuclear changes: nucleomegaly, round intranuclear inclusions with eosinophilic staining, and amphophilic inclusions separated from the thickened nuclear membrane by a clear halo, creating an “owl’s eye” appearance (circles). H‐E, 20x. (c) Hepatocytes with ballooning degeneration (arrows) due to severe acute edema. H‐E, 40x. (d) Foci of lobular necrosis (thin arrow), biliary pigment indicating cholestasis (thick arrow), and intranuclear viral inclusion (circle). H‐E, 40x. (e) Areas of hepatocyte regeneration. H‐E, 20x.

An eye fundus examination reported CMV findings such as active bilateral retinitis and cotton‐wool retinal infiltrates. To assess histologic and prognostic involvement, a liver biopsy was performed with findings of acute hepatitis associated with morphologic changes related to the viral cytopathic effect of CMV, with areas of changes related to eosinophilic infiltrate and adaptive hepatic regenerative response, indicating a good prognosis (Figure 2). The patient received supportive care including extracorporeal liver support and oral valganciclovir therapy. Following completion of the treatment, he demonstrated clinical and laboratory improvement, leading to hospital discharge with outpatient follow‐up. During subsequent outpatient visits, the patient remained asymptomatic with favorable clinical evolution and complete normalization of liver function tests.

## 3. Discussion

Severe acute liver injury represents deterioration of liver function characterized by increased transaminases, bilirubin, and coagulopathy without developing encephalopathy. The most frequent causes represent paracetamol intoxication, undetermined causes, hepatotropic virus infections, and drug‐induced liver injury (DILI) [1, 2].

This case represents an uncommon presentation of CMV‐induced severe acute liver injury with systemic dissemination in an apparently immunocompetent adult. CMV hepatitis typically occurs in immunocompromised hosts, making this presentation particularly noteworthy [3, 4]. The concurrent development of bilateral CMV retinitis alongside severe hepatitis with histologically confirmed cytopathic changes suggests a failure of normal immune surveillance mechanisms. In elderly patients, age‐related immunosenescence may predispose to viral reactivation and inadequate control of CMV replication, even in the absence of overt immunosuppression [7, 8]. Studies have shown that aging induces physiological alterations affecting immune function, with latent CMV infection exerting profound influence on the aging immune system [7]. Additionally, the presence of chronic comorbidities such as hypertension and dyslipidemia may contribute to a chronic low‐grade proinflammatory state that could compromise immune surveillance. This phenomenon could explain the unusual systemic involvement pattern observed in our patient, where CMV reactivation progressed from typically asymptomatic infection to acute liver injury despite no identifiable immunocompromising conditions.

The management of ALI and ALF is framed in the management of complications of liver dysfunction (encephalopathy, coagulopathy, and hyperammonemia), hemodynamic stabilization, and infection control, among others. When support treatment fails to compensate the clinical picture and as a measure to limit liver damage, liver support therapies artificial or biological are used. A key point independent of the etiology is to limit the perpetuation of the damage which is related to the uncontrolled hepatic and systemic inflammatory response and the increase in the expression of von Willebrand factor (VWF) which stimulates platelet activation and aggregation forming microthrombi and altering hepatic circulation; another pathophysiological determinant is macrophage activation which is triggered by the persistent inflammatory response and results in the differentiation of monocytes to giant macrophages that phagocytize the VWF multimer; these giant cells further aggravate the circulation in the hepatic sinusoids leading to ischemia and further liver damage [9]. In recent years, liver support therapies have been used as a bridge to liver regeneration or as a bridge to liver transplantation. Within these purification techniques, randomized controlled trials have shown that therapeutic plasma exchange (TPE) and DPMAS are the modalities associated with better survival and that they manage to reduce the inflammatory response by inhibiting monocyte and macrophage activity and eliminating VWF. They are currently two modalities with more evidence than classic therapies such as MARS and PROMETHEUS [9]. The liver regeneration observed in the pathological anatomy is represented by the presence of eosinophils that migrate toward hepatocytes; these recruited eosinophils secrete IL‐4 and subsequently IL‐4/IL‐13 signaling is activated which facilitates liver regeneration by stimulating the entry of inactive hepatocytes into the cell cycle, resulting in a favorable prognosis especially in patients with ALI with preserved hepatic functional reserve [10] as in the case of our patient.

## 4. Conclusion

The application of liver support therapies in the patient described, before the development of encephalopathy, is considered an important point in limiting the most feared complication with high mortality, such as endocranial hypertension, and also a key point was to limit the inflammatory response to avoid further liver damage while waiting to clarify the etiological cause and gain time until the start of specific treatment and achieve regeneration with limitation of damage. This case highlights the successful early recognition and prompt initiation of appropriate treatment using antiviral therapy and liver support therapy in managing severe CMV‐induced acute liver injury in an immunocompetent patient, potentially averting the need for liver transplantation.

## Consent

No written consent has been obtained from the patients as there are no patient identifiable data included in this case report.

## Disclosure

All authors approved the final version to be published.

## Conflicts of Interest

The authors declare no conflicts of interest.

## Author Contributions

Maiko Alejandro Tavera Díaz was responsible for conception, investigation, resources, formal analysis, and writing original draft of the case report. Annia Aguilar Loayza contributed to data collection and prepared the original draft, and Maiko Alejandro Tavera Díaz, Annia Aguilar Loayza, Carolina Beatriz Mejía Vargas, and Juan Fernando Mamani Ochoa revised it critically.

## Funding

No funding was received for this manuscript.

## Data Availability

The data used to support the findings of this study are included within the article.
